# Cast‐Molded Channelized Hydrogel Scaffolds With Stereolithography‐Printed Templates

**DOI:** 10.1002/bit.70007

**Published:** 2025-06-29

**Authors:** Chi Wang, Yingge Zhou

**Affiliations:** ^1^ State University of New York at Binghamton Binghamton New York USA

**Keywords:** channelized scaffold, hydrogel, stereolithography

## Abstract

Creating internal vascular networks within hydrogel scaffolds is crucial for providing the encapsulated cells with the necessary nutrients, oxygen, and metabolic exchange. Current methods for hydrogel scaffold fabrication face significant hurdles, including the challenge of forming sufficient internal channels, achieving precise scaffold geometry, and maintaining high cell viability, often compromised by the fabrication process and properties of the polymer materials used. Stereolithography (SLA) emerges as a promising 3D printing technique due to its exceptional precision, efficiency, and resolution, allowing for the creation of complex geometries with fine detail. This paper explores the application of SLA as a novel strategy to fabricate hydrogel scaffolds with interconnected small diameter channels, surpassing the capabilities of fused deposition modeling method to create templates. The encapsulated fibroblasts grown in the hydrogel scaffold containing channels showed significantly elevated cell viability compared to the ones without any channels. The capability of this SLA‐assisted strategy to create channel structures with encapsulated cells demonstrate significant potential for generating 3D artificial tissue composites with precisely controlled micron‐scale channels.

## 1 Introduction

As a branch of regenerative medicine, tissue engineering is increasingly recognized as a strategy for addressing organ donor shortages by producing artificial organs or tissues in vitro (Sharma et al. 2019; Hussain et al. 2022). These engineered constructs, such as blood vessels (Chandra and Atala 2019), bones (Guo et al. 2021), and skin (Zhang et al. 2020), are then transplanted in vivo once matured. Given its complexity, tissue engineering draws upon multiple disciplines, offering potential solutions to customize organs/tissues and mitigate the risk of immune rejection associated with traditional organ transplantations (Wang et al. 2022, 2021). In tissue engineering, hydrogels serve as versatile scaffold materials, as their in‐situ formability allows for precise encapsulation of drugs/cells, enabling minimally invasive surgical procedures and offering structural support as well as extracellular matrix mimicking abilities (Nezhad‐Mokhtari et al. 2019; Islam et al. 2016). With inherent biocompatibility, hydrogels promote cell proliferation and tissue integration, advancing regenerative medicine (Wang et al. 2020a; Han et al. 2020; Wang et al. 2020b). Vascularized scaffolds are essential in tissue engineering as poor vascularization hinders nutrient diffusion, waste removal, and jeopardizes cell viability (Heene et al. 2021). Scaffolds with channels resolve these issues by integrating channels inside the scaffold to mimic the blood vessels, therefore improving nutrients/waste transport, and aiding balanced cell distribution within the construct (Anthon and Valente 2022; Wu et al. 2023).

Current challenges in constructing channels within hydrogel scaffolds involve achieving precise channel geometry, maintaining structural integrity, and ensuring satisfactory cell viability (Akbari et al. 2020; Siddiqui et al. 2021; Boularaoui et al. 2020; Liu et al. 2017). Achieving these goals is complicated by an inherent conflict between cell viability and shape fidelity. Additionally, ensuring sufficient permeability for nutrient exchange is also challenging, and it directly impacts cell viability within the scaffold (Bomkamp et al. 2022). Current methods for fabricating perfusable channels in hydrogel materials could be summarized as direct printing and templating strategies. For instance, to realize three‐dimensional structures with microchannels, shell/core nozzles have been utilized to directly print concentrated alginate paste with aqueous poly(vinyl alcohol) (PVA) (Luo et al. 2012). Similarly, a coaxial approach may also be employed to fabricate 4D thermoresponsive channeled hydrogels (Podstawczyk et al. 2024). However, direct printing encounters challenges in achieving complex vascular structures, as the deposited ink may collapse without adequate support. Moreover, with the assistance of sacrificial bath, direct printing hydrogel may achieve complex geometries but it also suffers from the resolution and fidelity issue (Brunel et al. 2022; Wang and Zhou 2024a). On the other hand, templating strategy, however, stands out for its precise control over channel geometry as it could serve as temporary support, making it a popular choice. Fused deposition modeling (FDM) stands as the predominant technology employed alongside templating strategies for fabricating channels within hydrogel scaffolds, owing to its versatility in material selection. Utilizing sacrificial materials enables the creation of templates with intricate geometries, thereby facilitating the fabrication of highly complex interconnected channel networks as well as other complex structures within the scaffold, such as interconnected lattice like structure channel (Wang et al. 2024) and hierarchical channel structure (Feng et al. 2020).

Stereolithography (SLA) techniques have been shown to provide higher printing speed, resolution, and superior surface finish compared to FDM (Hesham et al. 2022; Grzeszczak et al. 2021). SLA aims to overcome the resolution limitations of FDM, which struggles to produce channel sizes below 800 μm. By enabling the fabrication of finer features, SLA allows for the creation of smaller, more biomimetic vascular structures such as capillaries within hydrogel scaffolds. In our study, we adopted a variation of traditional SLA method, called masked stereolithography (MSLA) printer. Unlike traditional SLA, which uses a laser to trace each layer, MSLA uses an LCD screen to cure entire layers at once, enabling faster prints with pixel‐level resolution. The process is aided by a liquid crystal display (LCD), which selectively blocks UV light to cure the resin solely in desired regions. This innovation enhances printing speed and precision control, surpassing traditional SLA method. While SLA has been utilized for direct fabrication of channeled scaffolds by incorporating photoinitiator substances into the hydrogel (Zhang and Larsen 2017; Xue et al. 2018), exposure to UV light may potentially compromise cell viability when cells were encapsulated in the hydrogel.

Thus, we propose a novel approach leveraging SLA's high resolution to fabricate a hydrogel scaffold with perfusable interconnected channel networks by cast molding with slow‐gelling hydrogel. The SLA is less commonly explored to fabricate templates in the tissue engineering area due to the material limitations. This study aims to fabricate micro‐to‐macro sized channels within hydrogel scaffolds by SLA templates with geometrical accuracy and biocompatibility. The hypothesis is that SLA‐printed templates‐created channels with improved shape fidelity will affect mass transfer and material release profile within the hydrogel scaffold, resulting in improved cell viability. To test the hypothesis, various characterization and comparisons between SLA and FDM‐assisted hydrogel scaffolds were conducted, biological validations such as biodegradation, cytotoxicity, and cell viability were also investigated.

## 2 Material and Experimental Methods

### 2.1 Solution Preparation

Sodium alginate (SA) powder was obtained from SIGMA‐ALDRICH, Co. (St. Louis, MO, USA,). Hydrolyzed type I and type III collagen (Col) powder was acquired from Doctor's Best Inc. (Irvine, CA, USA). Granulates of Delta‐Gluconolatone (GDL, Molecular weight:178.14) were sourced from Acros Organics, Co. (Belgium). Calcium carbonate (CaCO_3_, Molecular weight:100) powder was purchased from Thermo Fisher Scientific (Ward Hill, MA, USA).

Sodium alginate and collagen (SA‐Col) composite solutions were prepared by dissolving 1% (w/v) SA and 1% (w/v) Col in ultrapure water through magnetic stirring for 24 h at room temperature. The 0.45% (w/v) calcium carbonate (CaCO_3_) solution was made by adding CaCO_3_ powder in ultrapure water and stirring thoroughly before every use. Gluconic acid δ‐lactone (GDL) was added to CaCO_3_ solution at 0.8% (w/v) concentration and quickly mixed with SA‐Col solution. The CaCO_3_ and GDL solution was mixed with SA‐Col solution in a 1:1 ratio. Consequently, the final concentrations of SA and Col in the crosslinked hydrogel were both 0.5% (w/v).

### 2.2 3D‐printed Templates and Channelized Scaffolds Fabrication

**Figure 1:**
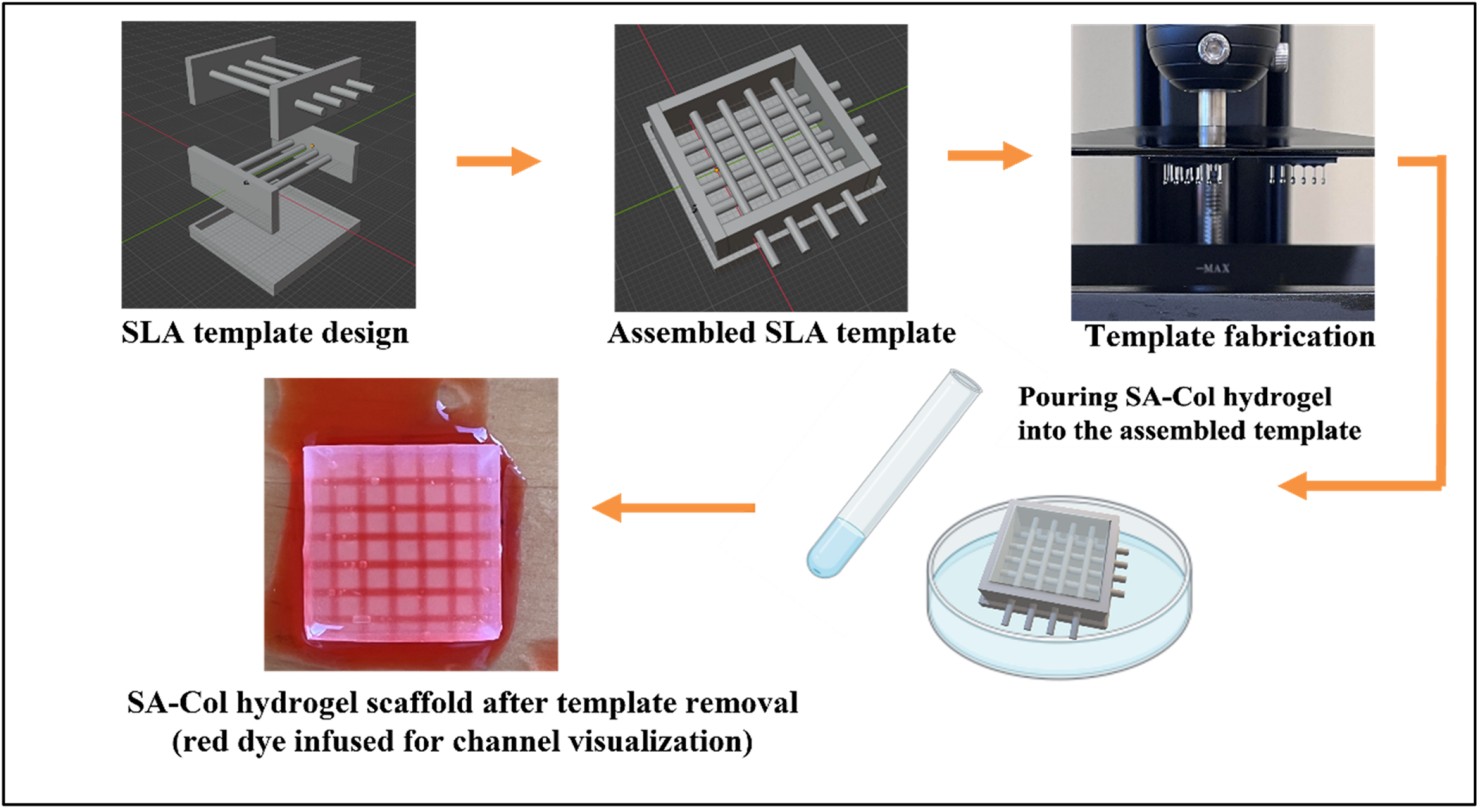
The fabrication process of the SLA template and the hydrogel scaffolds.

The 3D printed templates were prepared by SLA and FDM processes. The overall manufacturing process of SLA strategy is summarized in Figure 1. The FDM template utilized the identical designed STL file as SLA, it was printed by Prusa i3 printer with 0.25 mm nozzle and the silver PLA filament acquired from Ultimaker. First, five components of the template were designed using Blender with the desired bar diameter and densities. Second, the 3D model data was transformed either by Chitubox or Ultimaker Cura 5.3. Third, the template pieces were fabricated either by SLA or FDM using the following parameters. The SLA fabrication was carried out using the Elegoo Mars 3 Pro printer, manufactured by Elegoo Technology Co. (Shenzhen, China). We used water‐washable resin from Siraya Tech (San Gabriel, CA, USA), which, after complete curing, was confirmed to be biofriendly in the cell viability tests. The FDM printer is Prusa i3 MK3S with a 0.25 mm nozzle. SLA printing parameters are as follows: an exposure time of 3 s, a bottom layer exposure time of 30 s, and a layer height of 0.05 mm. Additionally, a tolerance compensation of 0.5 mm is applied to minimize overexposure at the bottom, which could otherwise lead to poor fitting of the parts. The optimized FDM parameters are as follows: the 0.2 mm nozzle, a printing speed of 30 mm/s, an infill density of 20%, and a layer height of 0.15 mm, temperature of 200°C. In terms of SLA fabrication, postprocessing steps are required. These include washing the residual resin from the printed templates using 70% ethanol and post‐curing them under UV light for 20 min. As shown in Figure 1, the four‐part templates were carefully assembled and secured by the bottom piece, into which the SA‐Col hydrogel was cast. Following complete hydrogel crosslinking, which occurred in approximately 15 min, the bottom piece was removed first, followed sequentially by the left, right, top, and final side pieces. This stepwise removal process was facilitated by the smooth surface of the SLA templates, which minimized adhesion and enabled precise scaffold extraction with minimal deformation. Consequently, the hydrogel scaffolds retained their intended geometry effectively, demonstrating the reliability of this method for producing geometrically accurate scaffolds.

### 2.3 Surface Hydrophilicity

The scaffold hydrophilicity test measured their ability to attract and retain water, and it is crucial for ensuring proper cell interaction and nutrient diffusion in biomedical applications. The surface hydrophilicity test on templates made from various materials using FDM or SLA was conducted, as it is crucial in determining whether hydrophilicity affects hydrogel flow and gelation in the template, potentially leading to air voids or gaps. The surface hydrophilicity of the scaffolds was assessed by measuring the water contact angles of these scaffolds at room temperature. Contact angle measurements were performed utilizing the Attension Theta Optical Tensiometer (Biolin Scientific, Finland). A consistent drop (5 μL) of distilled water was deposited onto the scaffold surface and a reference template surface using a fine needle. The progression of the droplet deposition and contact angle change process was recorded by the instrument and the contact angle was evaluated for 10 s and the average contact angle value was calculated.

### 2.4 Design of Channel Size and Density

To explore the effect of channel diameters and channel density on the final channel geometry, we made scaffold samples with three channel densities and three diameter sizes. The SLA fabricated template and corresponding hydrogel scaffolds were with different channel densities (2 × 2, 4 × 4, and 6 × 6) and diameters (400, 800, and 1600 µm). The channel diameter was measured horizontally and vertically for each cross‐section area of the channel. Three scaffold samples, each comprising four slices, were collected from every group, resulting in a total of 24 cross‐sectional areas of channels measured for each group. The cross‐sectional area of the channels was observed under the EVOS XL Core optimal microscope (Thermo Fisher Scientific Inc., Waltham, MA, USA). Both the channel diameter and circularity were measured using ImageJ (National Institutes of Health, Bethesda, MD, USA).

### 2.5 Release Profile

By studying material release profile, we can comprehensively validate the improvements in mass transfer within channelized scaffolds, investigating which designed factors effectively enhance the movement of essential molecules and thus support better cell growth. The dye‐loaded scaffolds with channels (abovementioned nine groups of diameters and densities) and without channels were immersed in 250 mL water at room temperature. At designated time points (5 min to 18 h), 1 mL of the surrounding water, where the dye had diffused from the hydrogel, was collected for colorimetric analysis. A glass rod was used to gently stir the liquid to ensure the even distribution of the released dye. Release profiles were determined using ImageJ processing with channel split and mean pixel intensity function.

### 2.6 SLA‐ Versus FDM‐Assisted Scaffolds

To conduct a comparative study between the FDM‐fabricated template and the SLA‐fabricated template, we opted to focus on a 6 × 6 grid with 1600 µm group. This decision was influenced by the limitations of our FDM printer, which struggles with fabricating templates featuring bars of 400 and 800 µm diameters. We compared the surface roughness of the hydrogel scaffolds, the precision of channel geometry, and the release profile between scaffolds produced using these two additive manufacturing techniques. Surface roughness was assessed by setting the nominal line and measuring the deviation of the surface profile at multiple points. Channel geometry precision encompassed measurements of channel diameter and circularity, which were then compared with both the design and the template sizes. The release profile was analyzed using the same methodology outlined in Section 2.5.

### 2.7 Biodegradation of Hydrogel Scaffolds

To investigate the in vitro biodegradation of the scaffolds and assess the potential influence of the scaffold channelization on degradation rates, two distinct groups were fabricated using equivalent volumes of hydrogel (1 mL). These groups included bulk scaffolds and channeled scaffolds. Subsequently, the scaffolds were immersed in Eagle's Minimum Essential Medium with l‐Glutamine (EMEM) (Quality Biological Inc, MD, US) and incubated at 37°C. At designated time intervals, the scaffolds were retrieved, air‐dried, and weighed. Degradation rates were determined by dividing the air‐dried weight of the scaffold at a specific time point by the initial air‐dried weight of the scaffold (at day 0). The SLA‐fabricated template for the channeled scaffolds was designed with 2 × 2 bars with diameters of 2 mm.

### 2.8 In Vitro Cytotoxicity

Gibco™ Human Dermal Fibroblasts, adult (HDFa) cells (Fisher Scientific, US) were cultured in Eagle's Minimum Essential Medium with l‐Glutamine (EMEM) (Quality Biological Inc, MD, US), and supplemented with 10% fetal bovine serum (FBS, Mediatech, CA, US). Cells were cultured in a 37°C, 5% CO_2_ incubator, with medium refreshed every 24 h, and subcultured once cell confluency surpassed 90%.

The in vitro cytotoxicity analysis of SA‐Col scaffolds was performed by MTT [3‐(4,5‐dimethyl‐2‐thiazolyl)‐2,5‐diphenyl‐2HO tetrazolium bromide] assays with a direct method, in which cells were directly contacted with the hydrogel. The 0.4 mL SA‐Col hydrogel was used to cover the bottom of the 24‐well plate and fully crosslinked. The cell density was 0.3 million/well. 1 mL of Eagle's Minimum Essential Medium with 10% FBS was added to each well plate and the medium was changed every 24 h. MTT assays were performed to determine the cell viability every 24 h. The principle of the MTT assay is based on the ability of viable cells to convert the yellow water‐soluble tetrazolium salt MTT into a purple formazan product. This conversion occurs primarily in the mitochondria of metabolically active cells. The well plates were replenished with 1 mL of fresh medium, followed by the addition of 0.1 mL MTT solution (0.833 mg/mL in phosphate buffer saline) to each well. After incubation for 4 h, the 1 mL isopropanol with 0.04 N HCl was added to each well and mixed thoroughly by repeated pipetting. The HCl converts the phenol red in medium to a yellow color that does not interfere with MTT formazan measurements. The isopropanol dissolves the formazan to give a homogeneous purple solution suitable for absorbance reading. The optical density was read using Synergy Neo2 Plate Reader (BioTek, Vermont, US) at 570 nm. The background wavelength was subtracted from the tested wavelength at 630 nm. The cells were seeded directly on the bottom of the plate with complete medium without hydrogel as positive control and set complete medium as blank. Cell viability was determined by the given formula: (OD_sample_ − OD_Blank_)/(OD_control_ − OD_Blank_) × 100. Each group includes three replicates per test condition, ensuring consistent sampling across all experimental groups.

### 2.9 Live/Dead Assay

To prepare a cell‐encapsulated SA‐Col scaffold, human fibroblast cells were detached from the culture flask with 0.05% trypsin (Mediatech, VA, US). Then the cells were resuspended in a SA‐Col solution before crosslinking. The UV‐sterilized SA‐Col, CaCO_3_ solutions, and GDL granulates were prepared and the SLA‐fabricated templates were properly post‐cured and sterilized by 70% ethanol, followed by additional sterilization with UV light. The cell‐mixed SA‐Col solution was combined with CaCO_3_ solution and GDL, and immediately cast into the assembled templates. The channels have a diameter of 3 mm and a length of 10 mm. The dimensions of the template are 1 × 1 × 1 cm cube. Each scaffold, with or without channels, obtained a final hydrogel volume of 1 ml. The cell density in each cell‐laden scaffold was set to 2 million cells/ml of hydrogel. After 10 min of hydrogel crosslinking, templates were removed and the cell‐laden scaffolds were immersed in 10 ml of culture medium within a cell culture flask and incubated in a 37°C, 5% CO_2_ incubator, with refreshed medium every 24 h.

Cell viability in the SA‐Col scaffolds was studied using a Live/Dead assay. At Days 1 and 7, the 3D scaffolds were cut in half, and the cut cross‐section that was not in contact with the medium was stained with calcein, AM, cell permeant dye as live cell indicator and BOBO‐3 Iodide as dead cell indicator. The cross‐sections were immediately examined for cell viability with a confocal laser scanning microscope (LSM 880; Carl Zeiss, Jena, Germany) using a FITC/Texas Red filter. The imaged cells were counted using ImageJ software. The ratio of live cells to the total number of cells was used as a metric of cell viability. Each group includes three replicates per test condition, ensuring consistent sampling across all experimental groups.

### 2.10 Statistical Analysis

Data were analyzed using Two‐Way ANOVA to assess the main effects of channel diameter and channel density, as well as their interaction on the outcome measures. Pairwise comparisons between the groups were performed using Tukey's Honest Significant Difference (HSD) test to determine specific differences between group means. Statistical significance was considered at *p* < 0.05, with results indicated in the figures by * for *p* < 0.05 and ** for *p* < 0.005.

## 3 Results and Discussion

Multiple physical, geometrical, and biological characterizations were conducted to validate the novel SLA‐assisted hydrogel scaffolds. The channel diameters and channel density incorporated in the hydrogel scaffolds varied, and these are classified into nine groups with three levels of channel densities (2 × 2, 4 × 4 and 6 × 6) and diameters (400, 800, 1600 µm). We also compared the scaffold properties between scaffold templates produced using SLA and FDM techniques. The assessment of biodegradation, hydrogel cytotoxicity, and cell viability within the scaffold will help evaluating the potential of this novel SA‐Col scaffold in tissue engineering applications.

### 3.1 Surface Hydrophilicity

**Figure 2:**
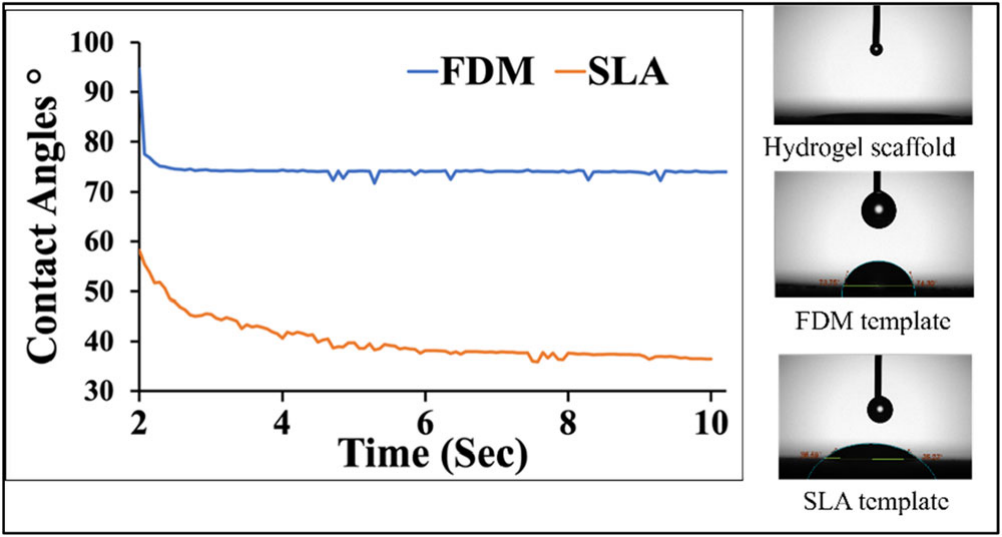
The water contact angle of hydrogel scaffolds, SLA template, and FDM template surfaces.

The mean water contact angles of SA‐Col scaffolds, SLA template, and FDM template at the end of the test (10 s) are shown in Figure 2. The water contact angle of the hydrogel scaffold is less than 15 degrees, which rapidly decreased upon contact with the hydrogel that makes it unmeasurable at the end of tests. This is expected with high water‐content of the hydrogel. Cells adhere more effectively to hydrophilic surfaces and increasing hydrophilicity of the scaffold may lead to better cell and tissue compatibility, enhanced transport of oxygen/nutrients into the scaffold, and additional cell recognition sites on the scaffold (Oh and Lee 2013).

The water contact angles were 38.75 degrees on the SLA template and 74.17 degrees on the FDM template. The results indicate that the SLA mold has a smaller water contact angle compared to the FDM‐printed PLA mold due to higher hydrophilicity of the photopolymer resin than PLA, a thermoplastic with relatively lower surface energy. Furthermore, our findings show that SLA prints have smoother and more uniform surface finishes, which enhance wettability, while FDM‐printed PLA displays a rougher texture with micro‐gaps that may trap air and increase the contact angle. If the template surface has higher hydrophilicity, it would likely promote better wetting and smooth spreading of the hydrogel solution instead of retraction upon cast molding of hydrogel (Gorin et al. 2022). The hydrogel would tend to adhere more readily to the surface of the template, resulting in enhanced geometry precision and completeness of the fabricated hydrogel scaffold.

### 3.2 Scaffold Channel Geometry

**Figure 3:**
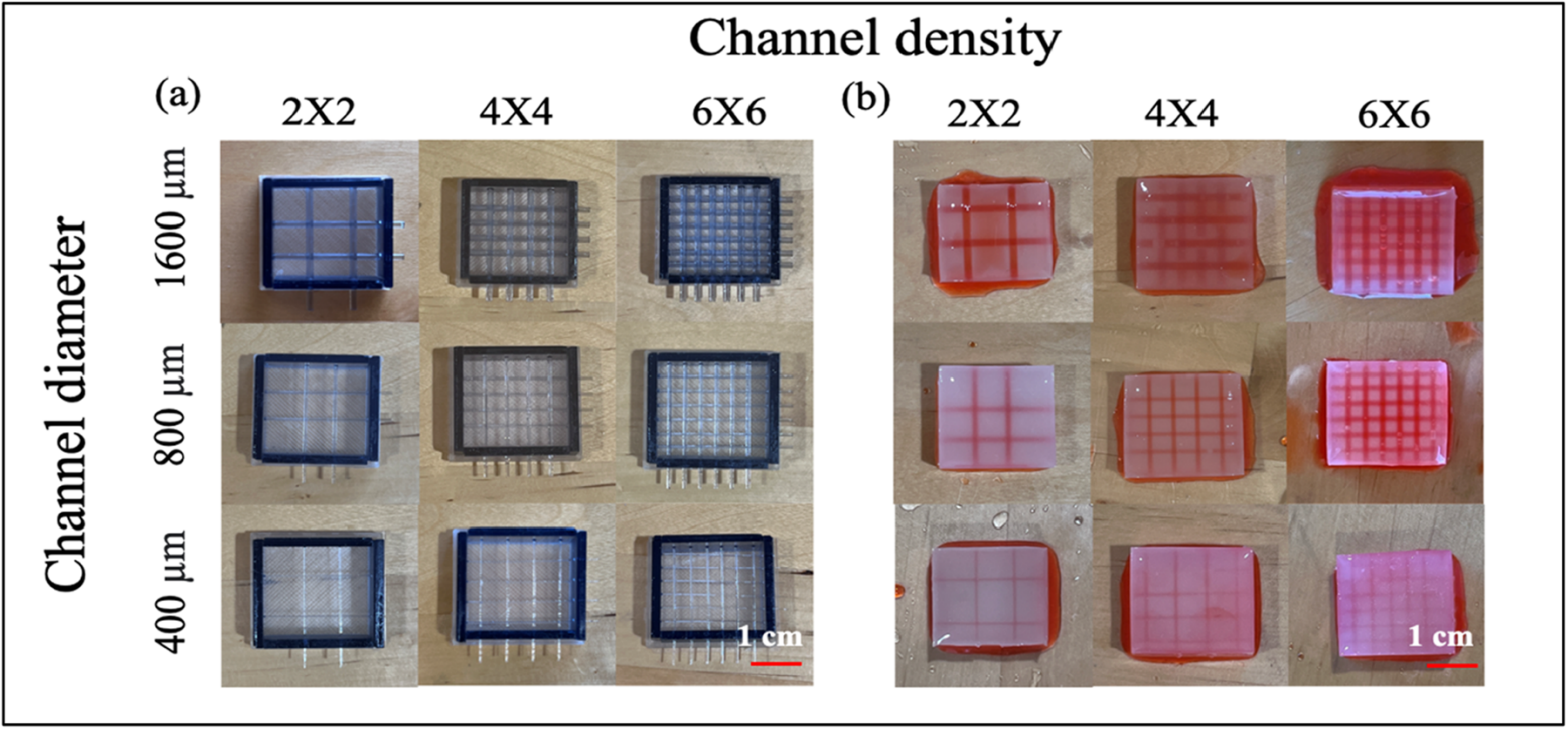
(a) Samples of SLA printed scaffold templates; (b) corresponding hydrogel scaffolds post‐template removal: Internal channel visualization enhanced with red dye infusion.

A channel geometry precision test was conducted to assess the shape fidelity of printed SLA templates and corresponding hydrogel channels. The impact of channel diameter and channel will also be discussed. Figure. 3 illustrates the SLA templates and corresponding hydrogel scaffolds, demonstrating various channel densities and diameters. Although the channel templates are positioned on different planes, the cast‐molded hydrogel scaffold successfully forms interconnected channels due to the precise assembly of the four‐piece templates. This interconnected structure is consistently achieved across all groups, even with channel sizes as small as 400 µm, as confirmed by dye infusion experiments. The hydrogel channels faithfully replicate the geometry of the 3D‐printed templates, indicating this fabrication strategy can achieve interconnected channels with various sizes and densities within the hydrogels.

**Figure 4:**
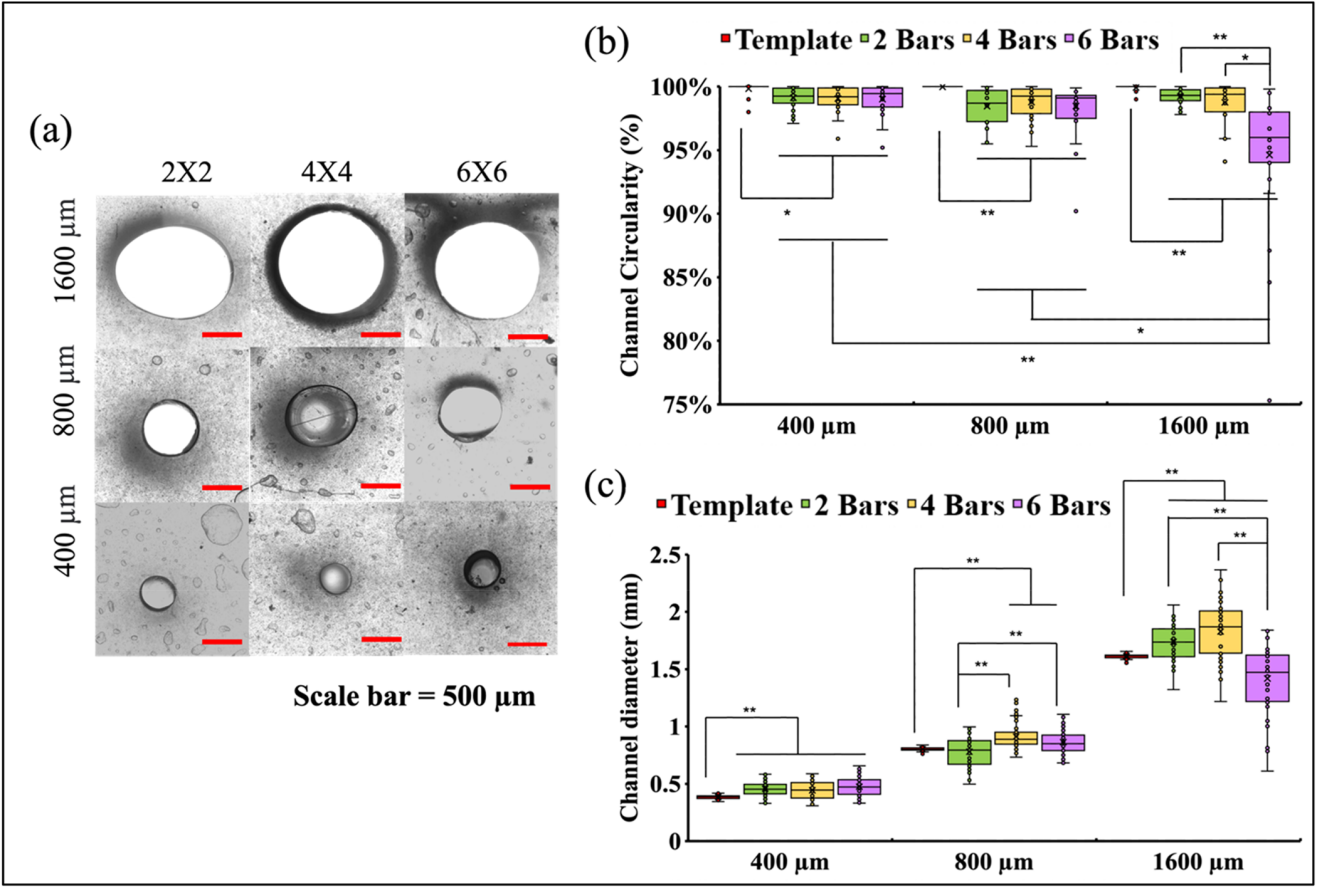
(a) Samples of cross‐section of channels in nine groups with various diameters and densities; (b) Channel circularity; (c) Channel diameter; * for *p* < 0.05 and ** for *p* < 0.005.

Figure 4a showcases nine cross‐sections of fabricated channels, with three diameter groups (400, 800, 1600 µm) and three density groups (2 × 2, 4 × 4 and 6 × 6). Figure. 4b summarizes the channel circularity among nine scaffold groups and the SLA templates. Figure 4c demonstrates the differences in channel diameters among all the groups and the printed SLA templates. Circularity analysis of hydrogel scaffold channels with varying diameters and densities revealed distinct trends. Channels with a diameter of 400 μm exhibited the higher circularity, while larger diameters, such as 1600 μm with the density 6 × 6, displayed the lower circularity. Conversely, larger diameters posed a higher risk of reduced circularity due to possible hydrogel collapse and deformation. Additionally, the effect of densities on channel circularity becomes more distinct in 1600 μm diameter groups, indicating interaction effects between channel size and density. Despite variations, circularity remained satisfactory overall, with mean values exceeding 95% in all groups. These findings underscore the scaffold's robustness in maintaining circularity, even under varying diameter and density conditions.

In diameter analysis, the SLA templates demonstrated highly aligned values with designed specifications. This can be arguably explained by the high screen resolution of 4 K and printing resolution at 35 microns in SLA printing. The channel diameter showed an increased variation when the channel size increased, with the 1600 μm diameter group displaying the lowest alignment with the design value. This deviation could be due to typical channel deformation caused by the weak strength and stiffness of the hydrogel. Factors such as gravity, flow‐induced shear stress, or solvent‐induced swelling of the hydrogel may contribute to this deformation (Dangla et al. 2010; Bian et al. 2009; Fernandez and Bausch 2009; Huang et al. 2012). Similarly, the effect of density on the channel diameter becomes more dominant in the 1600 μm group.

In summary, while smaller diameter channels maintained better alignment with predesigned diameter and circularity, larger diameters were prone to deviation, particularly with high channel densities. These findings underscore the correlation between the hydrogel macro‐porosity and channel geometrical precision, emphasizing the importance of model design and fabrication setup on achieving optimal scaffold geometry and integrity.

### 3.3 Release Profile

The release profile in scaffold‐based systems plays a pivotal role in the development and optimization of therapeutic interventions. The release profile refers to the pattern and amount of substance released from the scaffold over time, which indicates the efficacy of material exchange in cellular metabolism and nutrient transportation. It is crucial as it shows the efficiency and consistency of mass transfer, which is governed by diffusion, convection, and advection. The sufficient exchange of nutrients, waste and signaling molecules is essential for maintaining cell viability and function during long‐term cell culture. The objective of this test was to investigate the effects of interconnected channels on the release profile. It is hypothesized that a larger surface area to volume ratio will result in a higher amount of material released within the same period. In this case, we incorporated the same amount of liquid dye in the hydrogel scaffold during the fabrication and used it to produce the release profile of scaffolds with various surface‐volume ratio.

**Figure 5:**
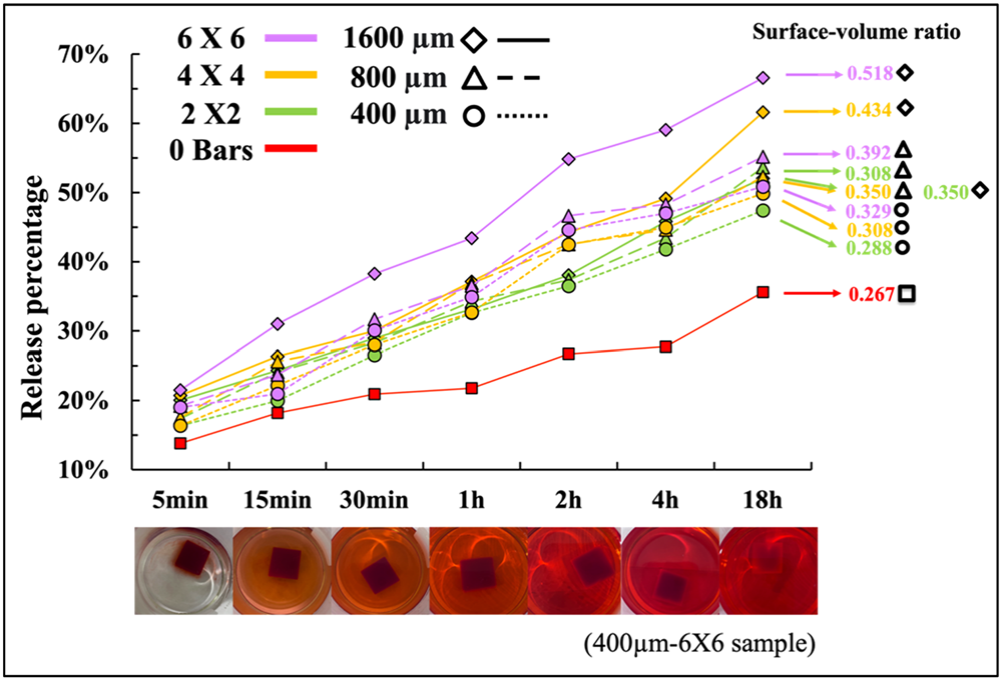
Release profile of scaffolds with different channel diameters and densities.

Figure 5 presents the results of all nine scaffold groups, alongside the control group, which contains no channel. It is observed that the 1600 µm 6 × 6 group exhibits the highest percentage of dye release among all groups, while the control group displays the lowest percentage of released dyes. These findings align with the scaffolds' surface‐volume ratios, indicating that higher surface‐volume ratio tends to result in increased material release and exchange over the same duration of time. Within 18 h, nearly all channeled scaffolds released at least 45% of the incorporated dyes, although this release rate may vary depending on the composition of the substances involved. It proves that the control of the channel diameter and densities would have a significant influence on the scaffold release profile.

This result aligns with Sadia's study that the inclusion of channels would accelerate the drug release rate, but the release pattern was also influenced by the width and length of the channel as shorter channels were more efficient at accelerating drugs despite having a comparable surface volume ratio (Sadia et al. 2018). In our samples, all channels have a length of 20 mm. Despite this, the 400 μm diameter groups continue to exhibit higher release rates compared to the control group, suggesting that none of the channels are obstructed. Additionally, Chew's group highlighted that additional factors such as scaffold degradation and porosity may also play crucial roles in determining drug release kinetics instead of surface‐volume ratio. Therefore, degradation assays were investigated to validate whether the presence of channels within the hydrogel scaffolds accelerates degradation rates, thus corroborating our findings.

### 3.4 SLA‐ Versus FDM‐Assisted Hydrogel Channels

**Figure 6:**
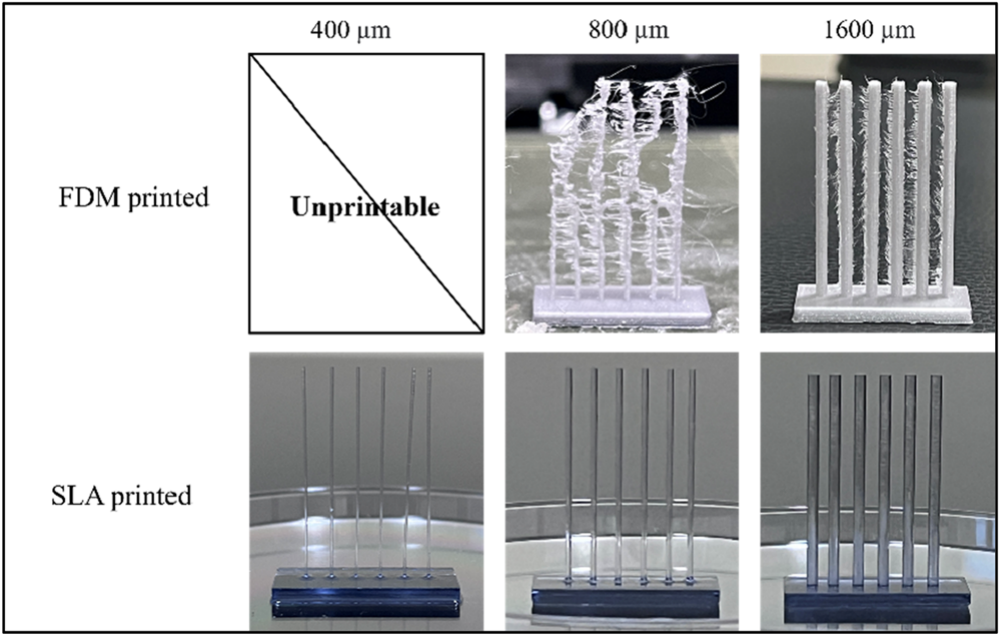
FDM and SLA printed templates with bar diameters ranging from 400, 800 and 1600 μm.

Compared to FDM, which is a widely used technique for fabricating templates used to create channels in hydrogels, SLA is less commonly utilized due to material limitations. In this study, we aim to compare the properties of channeled scaffold fabricated using FDM and SLA techniques, using the same STL file. The results indicate that SLA‐fabricated templates closely resemble the designed geometry with higher resolutions and precise features. Figure 6 showcased the printed templates fabricated by FDM and SLA printers. The 400 μm group could not be printed using our FDM printer (Prusa i3 MK3S) with a 0.25 mm nozzle. This is because FDM deposits melted PLA on the platform layer by layer, and typically, the diameter should be at least twice as large as the nozzle diameter. While the 800 μm group initially printed successfully, the FDM printer encountered difficulties when fabricating bars taller than 0.5 cm. This is due to the challenge of precisely depositing subsequent layers onto the previously printed layers, especially when the diameter is too small for the melted material to adhere accurately. In the FDM process, the bonding mechanism primarily involves the melting and solidification of thermoplastic materials, where the PLA filament is heated to the melting point and extruded. As each new layer is deposited, it partially melts the surface of the previous layer, allowing the layers to fuse upon cooling and solidification. On the contrary, the SLA printer was able to fabricate templates with diameters as small as 400 μm while maintaining superior quality. This is attributed to the SLA mechanism, which utilizes UV light projected by the LCD screen to solidify the resin, resulting in higher printing resolution. Ultimately, we selected the 1600 μm 6 × 6 group, as it could be successfully fabricated using both techniques.

**Figure 7:**
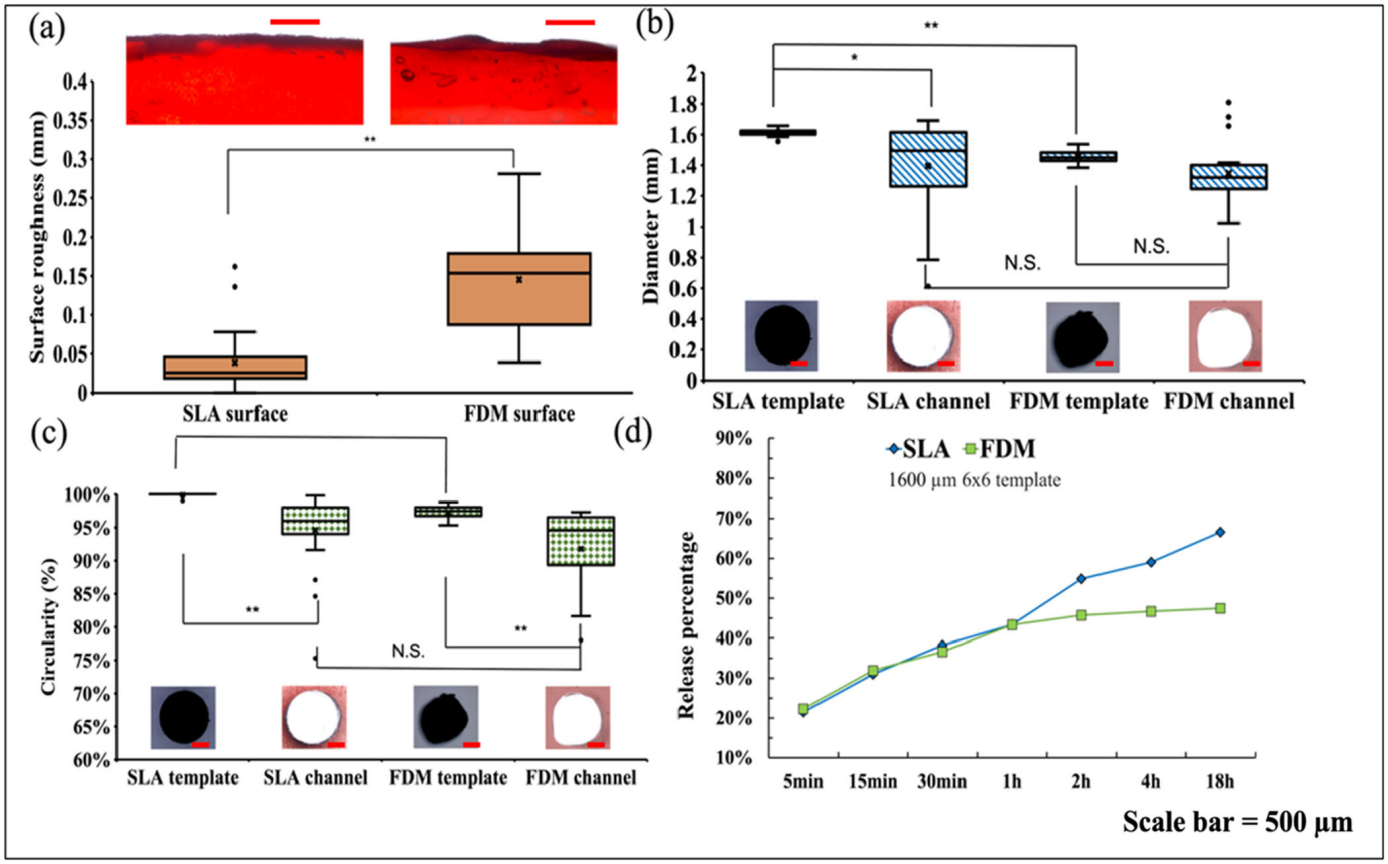
(a) Surface roughness; (b) Template and corresponding hydrogel channel diameter; (c) Template and corresponding hydrogel channel circularity; (d) Release profile of the two hydrogel scaffolds fabricated by SLA and FDM printed templates; * for *p <* 0.05 and ** for *p <* 0.005.

Figure 7a–d illustrates the comparative results between scaffolds created using templates from two different techniques. Figure 7a compares the surface roughness exhibited by hydrogel scaffolds fabricated via SLA and FDM methodologies. In the SLA group, surface irregularities were consistently controlled within 0.1 mm, yielding a mean roughness of approximately 0.02 mm, indicating a relatively uniform and smooth surface texture. Conversely, the FDM group displayed notably higher surface roughness, with mean values reaching 0.15 mm. This disparity can be attributed to the inherent characteristics of FDM printing, wherein the deposition of melted polymer follows a predefined path layer by layer, resulting in a discernible toolpath pattern that contributes to the observed roughness. Additionally, factors such as the nozzle temperature and viscosity of the melted polymer can influence the consistency of material deposition, further contributing to surface roughness (Mani et al. 2022; Ouazzani et al. 2023). In the other hand, SLA employs a photopolymerization process where liquid resin is cured layer by layer using ultraviolet light, which allows for more precise control for the feature and resulting in smother surface finishes. Surface roughness plays a crucial role in scaffold performance for tissue engineering applications. A rougher surface can enhance cell adhesion, proliferation, and differentiation by providing more surface area and topographical cues that mimic the natural extracellular matrix, especially for osteoblastic and chondrogenic tissue engineering (Zareidoost et al. 2012). On the other hand, smoother channel surface may help promote the medium circulation within the hydrogel networks, therefore affect the cell viability or drug release rate, which has been discussed in Section 3.3. Therefore, the differences in surface roughness between SLA and FDM‐printed scaffolds could significantly influence cell behavior and overall tissue integration, depending on the specific application.

Figure 7b and 7c present the diameter and circularity of templates by SLA and FDM as well as their corresponding channel geometry. The SLA templates closely adhere to the designed specifications, exhibiting a diameter of 1.6 mm and nearly 100% circularity. In contrast, the FDM templates display slight deviations, with a mean diameter of 1.4 mm and approximately 96% circularity upon fabrication. However, it is noteworthy that the fabricated hydrogel channels did not demonstrate significant differences in diameter between the two groups. This lack of discrepancy can be attributed to both sets of hydrogel channels experiencing collapse, likely due to insufficient support provided by the hydrogel material. Despite similar diameter outcomes, the circularity of the hydrogel channels differed noticeably between SLA and FDM groups. The SLA‐fabricated channels exhibited superior circularity, owing to the smoother surface characteristics of the SLA templates. Conversely, channels produced with FDM templates displayed surface irregularities, particularly evident in the zigzag pattern observed along the channel surfaces, as shown in Figure 7e. This phenomenon can be attributed to the less refined edge quality of the FDM template bars, as illustrated in Figure 6. The observed discrepancy in the diameter of FDM‐fabricated templates, approximately 0.2 mm smaller than intended, can be attributed to the inherent shrinkage tendencies of the PLA material during the FDM manufacturing process. The shrinkage phenomenon of PLA material during FDM has been documented by various research groups (Xinhua et al. 2015; Zharylkassyn et al. 2021). This shrinkage arises from the accumulation of residual stresses induced by nonuniform temperature gradients experienced during the repetitive heating and cooling cycles inherent to the FDM process (Li et al. 2018). Figure 7d illustrates the release profiles of two hydrogel scaffold groups. During the initial 1 h period, both groups exhibit a comparable release rate; however, the release rate of the FDM group gradually decelerates thereafter. This could be explained by the different channel geometries of the channels generated by SLA and FDM. Specifically, the SLA group's hydrogel channels boast larger average diameters and smoother surfaces, potentially facilitating the liquid permeation through the channels, thereby enhancing dye release into the surrounding water medium.

This observation underscores not only the geometric disparities between scaffolds fabricated using SLA and FDM templates but also their implications in tissue engineering applications. Geometric deviations in printed channel structures can significantly influence the biological performance of scaffolds. Deformation or inconsistency in channel size may disrupt controlled drug release by altering diffusion pathways, potentially leading to delayed or burst release profiles. Moreover, irregular geometry can hinder uniform cell seeding and migration, impair nutrient and oxygen transport, and compromise the mechanical stability of the scaffold (Mills et al. 2011). These factors collectively affect tissue integration and regeneration outcomes. Therefore, considering appropriate channel diameter and density within the scaffold is essential for ensuring both functional and biological performance in tissue engineering applications.

### 3.5 Biodegradation and In Vitro Cytotoxicity

**Figure 8:**
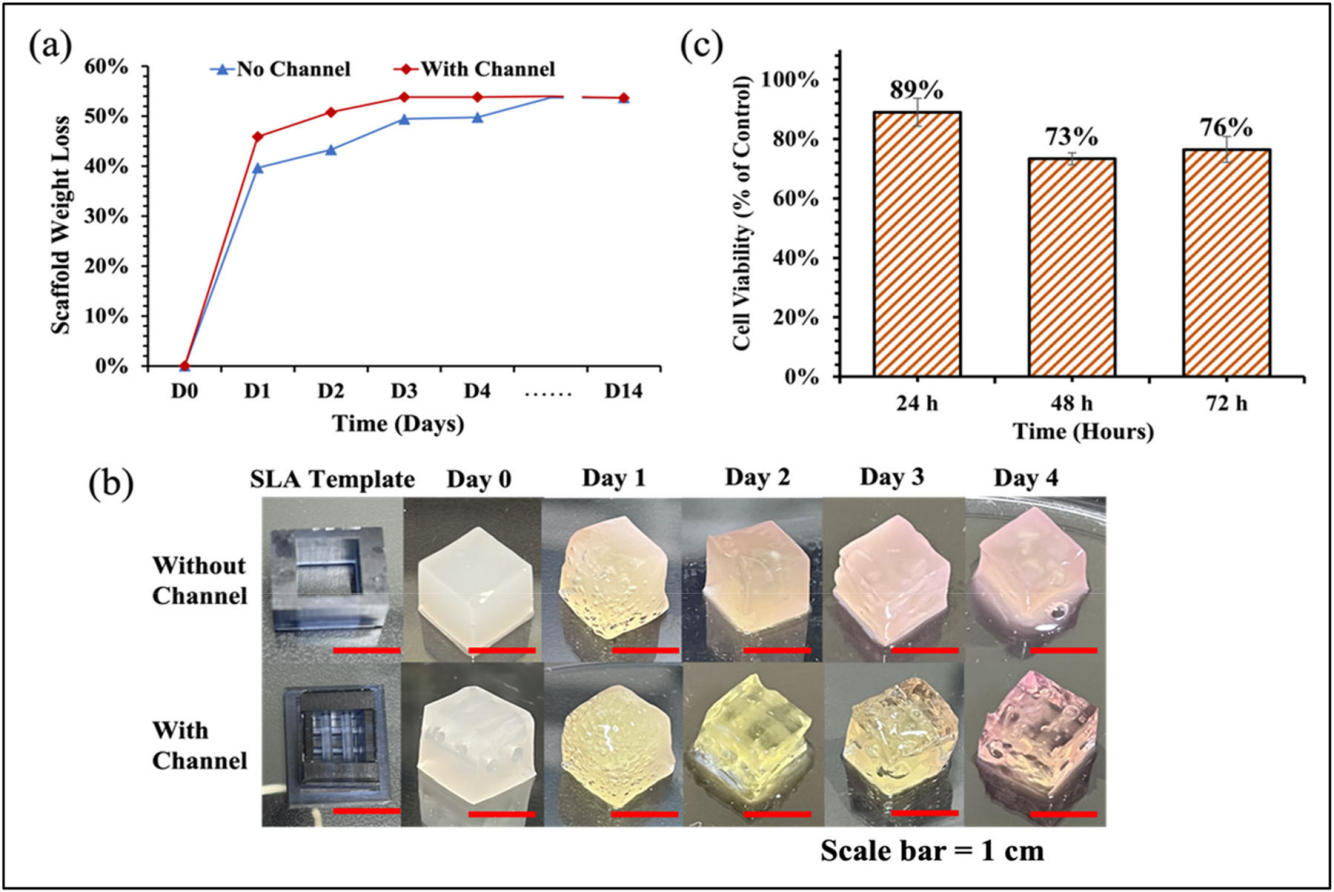
(a) In Vitro degradation of hydrogel scaffolds for 14 days; (b) Photos to degrading hydrogel scaffolds in first 4 days; (c) Cell viability with the hydrogel detected by MTT in 3 days.

The in vitro biodegradation of the SA‐Col hydrogel scaffolds was studied by incubating them in a complete medium at 37°C with the medium changed every 24 h, and weight‐loss of air‐dried scaffolds were monitored for 14 days. The dry‐weight loss and shape difference between hydrogel scaffolds also indicates the importance of channels. Hydrogel scaffolds could undergo degradation via erosion, hydrolysis, solubilization, and other biodegradation mechanisms, which involves complex dynamics (Gorgieva and Kokol 2012). On the other hand, the incorporated channels in the scaffold could influence the mass transfer, medium diffusion process, therefore differ the degradation process of the scaffold. Figure 8a presents that the channeled scaffolds lost weight faster than the solid scaffold in the initial few days. The degradation results showed similar trends between the two groups, both lost around 50% of their original weight on day 14. Most of the weight was lost in the first 2 days, and stabilized after day 3, meaning the fast degradation rate. Figure 8b also exhibits the shape fidelity of channelized and solid hydrogel scaffolds after incubation of different days. They both kept relatively good shape even after 50% of the weight had been lost, referring to good structure support for the cells during the degradation process. The nonenzymatic degradation of crosslinked SA‐Col hydrogel occurs primarily through hydrolysis and ionic exchange, where the crosslinked chains can be weakened by calcium ions dissolution into the medium and hydrolysis of SA and Col polymer bonds (Gao et al. 2009; Zhang et al. 2021a). The observed rapid degradation rate may be attributed to several factors. First, the significant initial decrease on the first day likely results from the release of residual crosslinker GDL, which initiates hydrolysis and subsequently accelerates the breakdown of the hydrogel matrix. The hydrolysis rate of the chemical bonds within the hydrogel is sensitive to pH; a lower pH promotes the hydrolysis of ester and amide bonds. This effect is visually confirmed in Figure 8b, where a color shift from yellow to pink over time as we change medium every day reflects an increase in pH, with yellow indicating a lower pH. Second, the relatively low concentrations of SA and Col compared to other studies contribute to the accelerated degradation rate. Finally, scaffold weight loss was measured in a fully dried state to eliminate any residual water in the channels, which could otherwise introduce error. This measurement approach likely accounts for the higher rate of weight loss compared to wet‐weight measurements. The final degradation rate is maintained at 50% at day 14, and other group also pointed out that ion‐crosslinked alginate is not naturally enzymatically degraded in mammals and months can be taken before it completely removed from implantation sites (Prang et al. 2006).

Figure 8c presents the results of hydrogel cytotoxicity using the direct method, which involves directly exposing cells to the material being tested to assess its cytotoxicity, unlike the indirect method that assesses cytotoxicity by exposing cells to extracts or leachates from the material. Cytotoxicity is the key study to detect the toxic effects of the proposed material and process used biomedical and tissue engineering fields. In our investigation, the viability of fibroblast cells attached to the hydrogel determined the cytotoxicity results. The cell viability was 89% after 24 h, and maintained above 70% after 48 and 72 h, therefore the prepared SA‐Col hydrogel was found safe and can be employed for tissue engineering applications. The favorable cytocompatibility of the SA‐Col hydrogel is suggested by Zhang's group too with above 90% survival rates with hUC MSCs and mouse fibroblasts cell line L‐929, meanwhile around 80% cell viability in encapsulated hydrogels after day 7 (Zhang et al. 2021b).

### 3.6 Encapsulated Cell Viability

**Figure 9:**
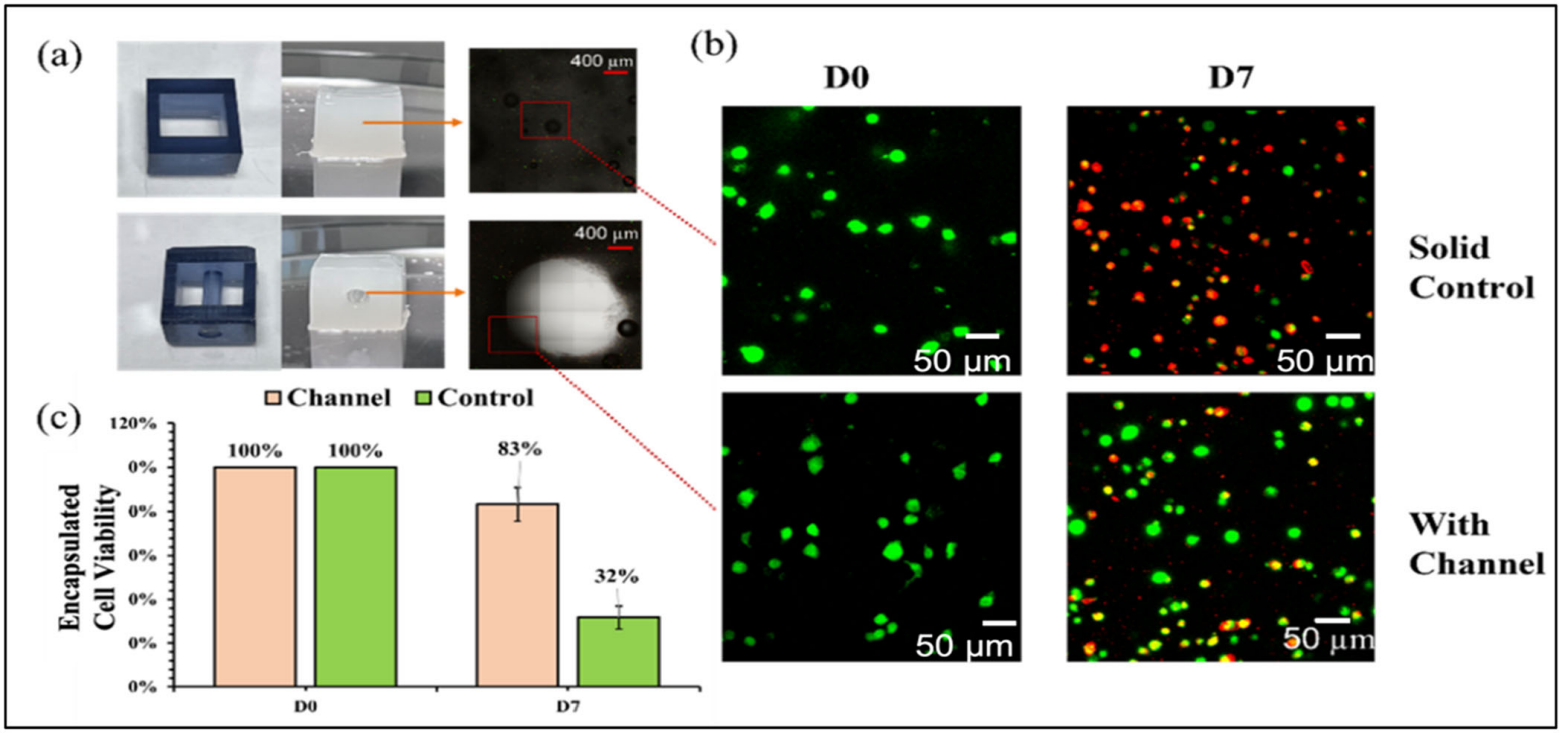
(a) SLA templates, hydrogel scaffold and confocal images of cross‐section area of scaffold channel and solid control part; (b) fluorescence images of cell viability encapsulated in the hydrogel scaffolds at day 0 and day 7; (c) cell viability.

Encapsulated cells in the hydrogel with template strategy may offer several benefits compared to direct bioprinting, primarily protecting cells from mechanical stress during the printing process. Additionally, bioprinting can struggle with issues such as overhangs, using a cast molding template can effectively avoid these problems, offering a more reliable method for creating complex structures. Despite the benefits, it is always challenging to keep the cell viable within the gels, especially for the thick hydrogel scaffolds. Therefore, it is essential to check the viability of cells encapsulated in hydrogel to ensure the nontoxicity of the SLA template, and whether cells could remain alive and functional overtime when encapsulated in the crosslinked SA‐Col hydrogel. Our hypothesis is that channelized scaffolds may enhance cell viability by facilitating more efficient mass transfer with the culture medium. Figure 9a present the SLA templates and hydrogel scaffolds with and without channels. Figure 9b presents the encapsulated LIVE/DEAD essay in the central region of the scaffold at day 0 and day 7. Figure 9c depicts the statistical findings of cell viability, revealing that channelized scaffold achieved approximately 83% cell viability at Day 7, whereas the solid control group exhibited only 32% cell viability. Both groups displayed 100% cell viability on day 0, indicating that the encapsulation process didn't immediately compromise cell viability. In our case, the pH level was carefully restricted around 7 to 7.6 to maintain the cell viability. The pH level was reset to 7.4 with medium changed every day. It is further demonstrated that the incorporation of channels in the scaffold promotes the exchange and mass transfer processes, thereby facilitating cell viability over the long run. Despite that collagen was incorporated, the reduced cell viability of channelized sample observed on day 7 could be due to several factors: (1) pH changes during the initial days may have impacted cell survival, (2) encapsulated cells are confined within the crosslinked hydrogel, which could restrict their activity and nutrient exchange, and (3) potential cell damage during sample preparation for confocal imaging, particularly during the halving process, may have contributed to lower viability. Addressing these issues may lead to improved cell viability in future experiments.

**Figure 10:**
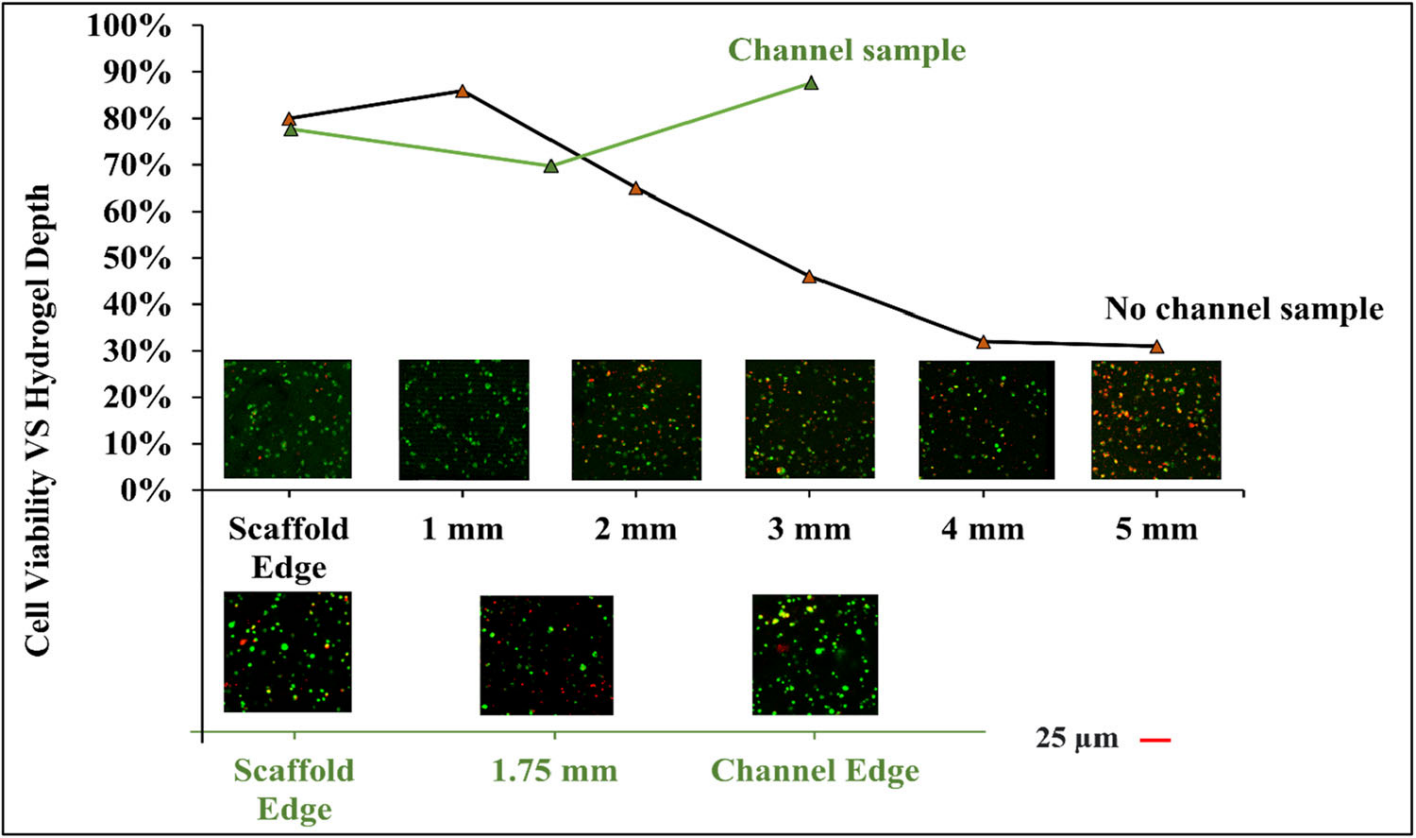
Encapsulated cell viability vs hydrogel depth (samples are 1x1x1 cm cube shape channelized & dechannelized hydrogel scaffolds).

On the other hand, to acknowledge the importance of channels within the scaffolds, we should also understand how cell viability decreases when hydrogel thickness increases. The diffusion of the oxygen and nutrients is often limited by distance of 100–200 microns, and cell death occurs more frequently in deeper regions of thick hydrogel scaffolds due to limited mass transfer (Ling et al. 2007; Radisic et al. 2005; Ishida‐Ishihara et al. 2022). Figure 10 further illustrates the relationship between encapsulated cell viability and depth of the hydrogel. The results show that without channels, the cell viability dramatically decreases as the hydrogel thickness increases beyond 1 mm. The viability at the very edge is not the highest because contact with tools and the petri dish during scaffold cutting might damage some of the cells at the periphery. For the channelized sample, the cell viability curve displayed a V‐shaped trend. This is because the edges of the scaffold are in contact with the medium, ensuring adequate nutrient and mass transfer. Despite the deepest region of the scaffold being approximately 1.75 mm, it maintained nearly 70% cell viability, thanks to the mass transfer occurring from both the scaffold and channel edges. These results could provide valuable insights for the scaffold design by considering cell viability in relation to channel densities and distributions. When utilizing this hydrogel material in the future, it is advisable to ensure that no part of the scaffold exceeds 2 mm in thickness. Additionally, channels should be incorporated at least every 2 mm to ensure maximum cell viability. Furthermore, the viability of cells in the deeper region of the scaffolds might be enhanced by adopting dynamic culture systems such as perfusion system (Ling et al. 2007), mechanical stimulation (Chen et al. 2020), and dynamic flow (Zhu et al. 2017), incorporating microchannels and pores and embedding growth factors can further facilitate encapsulated cell viability (De Witte et al. 2018).

## 4 Future Perspectives and Conclusions

In summary, this study demonstrates the potential to cast mold cell‐loaded SA‐Col hydrogel scaffolds with SLA templates. We conducted various assessments encompassing multiple physical, geometrical, and biological characterizations to ascertain the scaffold's potential in tissue engineering. Comparative analyses were performed between SLA‐assisted and FDM‐assisted scaffolds, focusing on geometry and biomedical properties. Our findings reveal that SLA‐assisted channelized scaffolds exhibit superior alignment with intended design parameters, accompanied by satisfactory cell viability within the scaffold. This underscores the potential of SLA techniques in advancing tissue engineering applications and addresses a crucial gap in utilizing SLA templates for such purposes. Looking ahead, enabling more complex channel geometries may involve the use of sacrificial materials, such as water‐soluble resins, which can be dissolved after fabrication to create versatile 3D internal structure within the hydrogel scaffolds. Alternatively, multi‐part mold designs could facilitate the removal of internal structures without damaging the surrounding matrix. For example, in a separate study, we developed an SLA‐printed mold that could be split into five interlocking components to fabricate an array of upright 3D vascular structures of varying sizes (Wang and Zhou 2024b). These strategies have the potential to significantly broaden the method's applicability to more physiologically relevant architectures in tissue engineering. Secondly, investigating long‐term cell behavior and enhancing scaffold degradation through adjustments in materials and crosslinkers would offer valuable insights into the scaffold's performance in vivo. Besides, to accelerate the SA hydrogel degradation rate, oxidizing SA to promote the hydrolysis or adopting enzymatically degradable SA crosslinker could be applied (Lueckgen et al. 2018, 2019). Last, while higher sodium alginate concentrations can reduce cell viability due to increased stiffness, adjusting the hydrogel formulation, such as reducing collagen from 1% to 0.5%, can help improve shape fidelity in some cases. This trade‐off might be acceptable to achieve balance between structural integrity and cell viability.

In conclusion, this study sets a foundation for leveraging SLA techniques in tissue engineering and lays the groundwork for continued advancements in scaffold design and applications. Based solely on cell viability results, the findings suggest that the SLA template did not show an intense negative effect on cell viability and is more suitable for applications requiring high‐resolution structures like capillaries due to its superior printing precision and improved biomimicry. In contrast, FDM's broader material compatibility allows for the use of biocompatible and biodegradable materials, which could be advantageous in minimizing toxicity and enabling the fabrication of complex interconnected 3D structures within hydrogel, though typically with larger channel sizes, through the use of sacrificial materials. By addressing future research directions, we aim to contribute to the ongoing evolution of scaffold‐based tissue engineering technologies.

## Ethics Statement

The authors have nothing to report.

## Conflicts of Interest

The authors declare no conflicts of interest.

## Data Availability

The data that support the findings of this study are available from the corresponding author upon reasonable request.
